# U.K. Community Earth System Modeling for CMIP6

**DOI:** 10.1029/2019MS002004

**Published:** 2020-09-21

**Authors:** Catherine A. Senior, Colin G. Jones, Richard A. Wood, Alistair Sellar, Stephen Belcher, Albert Klein‐Tank, Rowan Sutton, Jeremy Walton, Bryan Lawrence, Timothy Andrews, Jane P. Mulcahy

**Affiliations:** ^1^ Met Office Hadley Centre Exeter UK; ^2^ NCAS, School of Earth and the Environment University of Leeds Leeds UK; ^3^ NCAS, Department of Meteorology University of Reading Reading UK; ^4^ Department of Computer Science University of Reading Reading UK

## Abstract

We describe the approach taken to develop the United Kingdom's first community Earth system model, UKESM1. This is a joint effort involving the Met Office and the Natural Environment Research Council (NERC), representing the U.K. academic community. We document our model development procedure and the subsequent U.K. submission to CMIP6, based on a traceable hierarchy of coupled physical and Earth system models. UKESM1 builds on the well‐established, world‐leading HadGEM models of the physical climate system and incorporates cutting‐edge new representations of aerosols, atmospheric chemistry, terrestrial carbon, and nitrogen cycles and an advanced model of ocean biogeochemistry. A high‐level metric of overall performance shows that both models, HadGEM3‐GC3.1 and UKESM1, perform better than most other CMIP6 models so far submitted for a broad range of variables. We point to much more extensive evaluation performed in other papers in this special issue. The merits of not using any forced climate change simulations within our model development process are discussed. First results from HadGEM3‐GC3.1 and UKESM1 include the emergent climate sensitivity (5.5 and 5.4 K, respectively) which is high relative to the current range of CMIP5 models. The role of cloud microphysics and cloud‐aerosol interactions in driving the climate sensitivity, and the systematic approach taken to understand this role, is highlighted in other papers in this special issue. We place our findings within the broader modeling landscape indicating how our understanding of key processes driving higher sensitivity in the two U.K. models seems to align with results from a number of other CMIP6 models.

## Key Points

- The United Kingdom has developed its first community Earth system model and delivered a traceable hierarchy of models to CMIP6
- We applied a process‐based evaluation strategy in model development but chose not to use historic trends or measures of climate response
- The U.K. models exhibit higher climate sensitivity than seen in CMIP5 arising in part from more positive cloud feedbacks

## 1 Introduction

The HadGEM models of the climate system developed at the Met Office Hadley Centre (MOHC) and their predecessors are widely recognized as world‐leading contributions to the international ensemble of climate models. These models have been part of the Coupled Model Intercomparison Project (CMIP Meehl et al., 2005) from its first incarnation (CMIP1) to CMIP5, and projections from them have been included in every Intergovernmental Panel on Climate Change (IPCC) scientific assessment report. Versions of these models are also used for the U.K. Climate projections for which the world's first perturbed physics ensemble was developed (Murphy et al., 2004). Over this time, the MOHC models have also become increasingly used for research within the U.K. academic community, supported by the Joint Weather and Climate Research Programme (JWCRP), a partnership of the U.K. Natural Environment Research Council (NERC) and the Met Office. The JWCRP has for some time supported cooperative development between the Met Office and NERC Research Centres for specific climate model components and parameterizations. These include Ocean model development (Storkey et al., 2017), land surface model development (Walters et al., 2017), sophisticated parameterizations of aerosols (Mann et al., 2010), atmospheric chemistry (Morgenstern et al., 2009), ocean biogeochemistry (Yool et al., 2013), and ice sheets (Cornford et al., 2013). In addition, NERC Centres and U.K. universities, alongside other international partners making use of the Met Office Unified Model (UM), provide substantial evaluation of the MOHC models that feeds directly into model development. Clear examples of this work are the joint Process Evaluation Groups (PEGs) which form part of the Global Atmosphere (GA), Ocean (GO), and Sea ice (GSI) component model development cycles (e.g., Rae et al., 2015; Storkey et al., 2017; Walters et al., 2017). The PEGs have many academic members, and some are led by universities or UM partner institutions. The recognition of the increasing commitment of the NERC Research Centres and U.K. universities to the UM system led to the first discussions on how to jointly develop a second‐generation Earth system (ES) model that was targeted at the sixth‐generation Coupled Model Intercomparison Project (CMIP6, Eyring et al., 2016).

## 2 A Joint Development and Delivery Team

A core group for the development of a joint U.K. ES model (UKESM1) funded by both the Met Office and NERC was established in 2013, with NERC contributions led by the National Centre for Atmospheric Science (NCAS). The group is dispersed across nine locations, at the Met Office and NERC Centres (NCAS, BAS, BGS, CEH, CPOM, NCEO, NOC, and PML—see glossary for details) although a critical mass of jointly funded members sit within the Met Office. This facilitates efficient implementation of new model developments into the technical infrastructure of the UM system and strong engagement with the Met Office seamless physical (Atmosphere‐Ocean‐Ice‐Land [AOIL]) model development. This ensures that cycles of AOIL model development take account of any new dynamics, physics, or resolution changes in the physical model that might affect the ES model by impacting performance on long time scales or on components of the ES (e.g., through changes to hydrology that would influence carbon or nitrogen sources and sinks). Within the Met Office, new coupled AOIL UM releases are tested and evaluated from short‐range coupled Numerical Weather Prediction (NWP) time scales through seasonal hindcasts to multicentury present‐day (PD) simulations. In parallel these test versions are used in the development cycle of the ES model to enable a two‐way feedback process. The colocation of the UKESM core group and the Met Office seamless model development teams has enabled navigation of this complex landscape with maximum efficiency. Nevertheless, there have been significant challenges to deliver a model that satisfies the very diverse user requirements across this broad range of applications.

The Met Office, NERC Centres, and U.K. universities also collaborated on the data workflow and deciding the level of U.K. participation in the CMIP6 Model Intercomparison Projects (MIPs). Full details about the implementation of the models for the experiments, including treatment of CMIP6 forcing data and technical steps taken to ensure scientific robustness and reproducibility, are presented in Sellar, Walton, et al. (2019). The data workflow will be described elsewhere but depended on systems for developing CMIP compliant data developed in the Met Office and deployed by both Met Office and NERC staff, with all data flowing to an Earth System Grid Federation data node hosted by the NERC Centre for Environmental Data Analysis (CEDA).

## 3 Seamless Model Development

The UKESM project builds on the seamless approach to physical model development utilized by the Met Office for many years (e.g., Brown et al., 2012; Senior et al., 2010) A single model trunk is developed and tested across NWP, seasonal, and climate time scales. Final model configurations for each system requirement, including resolution and limited aspects of the physics, may differ (“branch” from the trunk) at given points in time dependent on final performance tests. However, all future development reverts back to the trunk baseline, and necessary changes made in the branches are either subsumed into the trunk going forward or abandoned as further development renders them no longer necessary or desirable.

The trunk systems are routinely documented for the atmosphere (GA), ocean (GO), sea ice (GSI), and land (GL) model components for each standard release. The ES development has followed a parallel trunk system, picking up the latest physical model versions at relevant points. If analysis of ES performance uncovers an issue with components of the physical system, this is then fed back into the model development cycle for the relevant physical component. Most initial physical model testing is done in sea surface temperature (SST)‐driven Atmosphere‐Land configurations (Atmospheric Model Intercomparison Project [AMIP] runs), typically at lower resolutions (∼60–90 km), and in surface flux‐driven ocean‐only configurations. NWP case studies at higher resolutions (∼40 km) and century‐long coupled AOIL simulations (mainly at ∼90 km atmospheric resolutions, occasionally at ∼60 km) are also carried out when significant new physics packages are developed.

The result of our most recent coupled AOIL testing was the physical coupled model, HadGEM3‐GC3.1 (a branch from the trunk model HadGEM3‐GC3), which constitutes the physical model core of UKESM1 (see also section 4). The additional ES components that take HadGEM3‐GC3.1 to UKESM1 were initially developed and tuned in uncoupled model configurations, with the final parameter values from those tests being used as the start point for testing the fully coupled UKESM1. For example, the values used for HadGEM3‐GC3.1 were the initial values for UKESM1's physical parameters at the beginning of the coupled testing. For computational reasons, a significant fraction of the development and tuning of the coupled biogeochemical cycles in UKESM1 was carried out using a model configuration with prescribed atmospheric chemistry (referred to as UKESM1‐CN). In parallel to this, coupling and tuning of the interactive trace gas chemistry scheme (UKCA, Archibald et al., 2019) with the GLOMAP‐mode aerosol scheme (Mulcahy et al., 2019) and relevant atmosphere and land physical parameterizations was carried out using the atmospheric component of HadGEM3‐GC3.1, GA7.1, with interactive chemistry enabled. Once the coupled biogeochemical cycles (in UKESM1‐CN) and the atmospheric chemistry‐aerosol processes (in GA7.1) were performing acceptably, this updated interactive chemistry scheme was activated in UKESM1‐CN, leading to the full configuration of UKESM1. Some further minor tuning was then performed before the final spin‐up, and then CMIP6 piControl runs were performed. Further details of the UKESM1 development process can be found in Sellar, Jones, et al. (2019) and the procedure followed to spin‐up the preindustrial (PI) control state of UKESM1 in (Yool et al., 2020).

The physical model, HadGEM3‐GC3.1, was applied across the CMIP6 MIPs at two resolutions: HadGEM3‐GC3.1‐MM (N216 [∼60 km], L85; ORCA025, L75; Menary et al., 2019; Williams et al., 2018) and HadGEM3‐GC3.1‐LL (N96 [∼90 km], L85; ORCA1, L75; Kuhlbrodt et al., 2018). The HadGEM3‐GC3.1‐LL and HadGEM3‐GC3.1‐MM resolutions were also used in HighResMIP, along with an even higher resolution version, HadGEM3‐GC3.1‐HH (N512 [∼25 km], L85; ORCA083, L75 Roberts et al., 2019). Due to the increased computational cost associated with the interactive biogeochemical cycles and full atmosphere chemistry, it was only feasible to run UKESM1 in CMIP6 at the same (N96, ORCA1) resolution as HadGEM3‐GC3.1‐LL. No additional tuning of the radiative balance of the physical modeling core was undertaken across the two resolutions or on the introduction of the ES components. This results in the two HadGEM‐GC3.1 models allowing an analysis of the benefits from increased (atmosphere and ocean) model resolution on simulated climate processes and feedbacks. Comparison between UKESM1 and HadGEM3‐GC3.1‐LL allows an assessment of the role of increased (ES) process complexity on coupled processes and feedbacks. Some initial results from this analysis, highlighting their impact on processes driving climate sensitivity, are documented in Andrews et al. (2019).

For CMIP6 we always planned to make contributions with both UKESM1 and HadGEM3‐GC3.1‐LL, the latter being the physical model core of UKESM1; we therefore developed as many of the cross‐process and cross‐domain couplings in UKESM1 to be based on internally predicted model variables. This contrasts with HadGEM3‐GC3.1‐LL (and other physical climate models) where numerous coupling fields, such as vegetation cover, seawater dimethylsulfide (DMS), or atmospheric chemical oxidants, are prescribed as time‐invariant fields, often based on observations. Following this model development procedure, of maximum internal prognostic coupling in UKESM1, increases the number of potential future feedbacks we can simulate with the model. At the same time, the increased degrees of model freedom enhances the risk of coupled biases developing. As a general rule, we included a new prognostic coupling in the model if (i) it was considered to have the potential to impact the the overall ES response to future scenario forcing, and (ii) the impact of including the feedback was neutral to positive, or only marginally negative, on the performance of the model when evaluated against relevant observational metrics. As a concrete example, in HadGEM3‐GC3.1 seawater DMS is prescribed from an observation‐based climatology (Lana et al., 2011), and the DMS emitted into the model atmosphere is oxidized to SO_4_ to act as cloud condensation nuclei, using prescribed and time‐invariant atmospheric oxidants. This means potential future changes in seawater DMS or the oxidizing capacity of the atmosphere cannot influence cloud and radiation responses in HadGEM3‐GC3.1. By contrast, in UKESM1 seawater DMS is predicted by the ocean biogeochemistry component of UKESM1 following the parameterization of Anderson et al. (2001). This seawater DMS is emitted into the model atmosphere where its oxidation to SO_4_ aerosol occurs through interaction with predicted oxidant fields that are, themselves, depleted through the oxidation process. This allows potential future changes in either or both seawater DMS and the oxidizing capacity of the atmosphere to influence the radiative properties of the clouds in UKESM1 and thus the overall climate response. A range of prognostic couplings such as those described here for DMS have been implemented in UKESM1, making it one of the most process complete and internally consistent ES models available today. More details on the development and tuning of key couplings in UKESM1 can be found in Sellar, Jones, et al. (2019) and Mulcahy et al. (2019).

Papers in this special issue describe results from both the physical models HadGEM3‐GC3.1 ‐LL and ‐MM and from UKESM1. Table 1 documents the choice of model configurations and resolutions for each of the MIPs in which U.K. models are engaged. The traceability of the family of U.K. models for CMIP6 is an important characteristic of our model development process and gives us a hierarchy of models and MIP experiments exploring both resolution and complexity space.

**Table 1:** U.K. Model Configurations Used for the DECK and MIPs in CMIP6

Model Configuration	Resolution	CMIP6 contributions
HadGEM3‐GC3.1‐LL (Kuhlbrodt et al., 2018)	N96, L85 ORCA 1°, L75	DECK, HIST, ScenarioMIP, CFMIP, DAMIP, FAFMIP, HighResMIP, LS3MIP, PMIP, RFMIP
HadGEM3‐GC3.1‐MM (Williams et al., 2018)	N216, L85 ORCA 0.25°, L75	DECK, HIST, ScenarioMIP, DCPP, GMMIP, HighResMIP, OMIP, PAMIP
UKESM1 (Sellar, Jones, et al., 2019)	N96, L85 ORCA 1°, L75	DECK, HIST, ScenarioMIP, AerChemMIP, C4MIP, CDRMIP, GeoMIP, ISMIP6, LUMIP, OMIP, PMIP, RFMIP, VolMIP, ZECMIP
HadGEM3‐GC3.1‐HH (Roberts et al., 2019)	N512, L85 ORCA 0.083°, L75	HighResMIP

## 4 Model Evaluation and Tuning

HadGEM3‐GC3.1 and UKESM1 have been developed and evaluated to offer improvements across as large a range of metrics as possible, with a focus on the mean climate and variability of the past few decades for which we have a huge range of observations available (much larger than the very restricted number of quantities which are observed further back in time). For the recent period the standard evaluation package for the physical model includes around 100 different variables considered important in the atmosphere, ocean and land, assessed both individually and combined in ∼500 assessment metrics. As a result, the models perform well for these variables (in terms of the simulation closely replicating reality and in comparison to contemporary CMIP5 models), as detailed in papers in this special issue (Kuhlbrodt et al., 2018; Menary et al., 2019; Sellar, Jones, et al., 2019; Williams et al., 2018).

Progress includes, but is not limited to, an improved representation of clouds, improved ocean stratification, improved representation of Arctic sea ice seasonal variability, and better simulation of the position of the jet stream. A focus on long‐standing systematic biases through the PEGs led to specific work being done during the development of GA7 (the GA component of HadGEM3‐GC3) to address the warm surface ocean bias in the Southern Ocean—a common problem in contemporary GCMs. Changes that improve this bias (e.g., representation of mixed‐phase cloud) have also been identified as key contributors to the increased climate sensitivity seen in the U.K. CMIP6 models compared to predecessor versions (Bodas‐Salcedo et al., 2019). An additional valuable evaluation tool that we can utilize because of the seamless nature of the UM is the Rodwell and Palmer (2007) method of NWP‐verification to evaluate new fast‐physics processes included in the HadGEM3‐GC3.1 and UKESM1 models (Williams et al., 2020). This approach finds that NWP skill scores are consistently improved by the inclusion of the key atmospheric changes that drive differences in climate sensitivity from GA6 to GA7 (Bodas‐Salcedo et al., 2019), providing an independent assessment of the improved physical basis of GA7.

The U.K. modeling community took the decision not to use any forced climate change simulations in developing the HadGEM3‐GC3.1 and UKESM1 models. By this we mean no historical (1850 to PD), 1%CO_2_ or abrupt4xCO_2_ experiments (Eyring et al., 2016) were used in the model development and tuning. As a result, the ability of the model to simulate the (observed) historical climate evolution, as well as its transient climate response (TCR) and effective climate sensitivity (EffCS), are all emergent properties of the model system. Model development and tuning were based on a mixture of AMIP runs and coupled runs with fixed PI or, additionally for HadGEM3‐GC3.1, PD (Year 2000) forcings, as well as a limited number of effective radiative forcing (ERF) experiments (discussed later). Comparison of control runs (either PI or PD) with observations taken from typically the past few decades is problematic in principle, but the focus of such work was to minimize large biases which were greater than the difference between PI and PD climate states (Menary et al., 2019; Williams et al., 2018). Thus, there is an underlying assumption that model mean biases and the response to historical forcings are to some extent independent. There are clearly risks associated with having no prior knowledge of the model's response to forced climate change—the biggest being that the resulting simulated climate evolution over the observed period (e.g., of global mean surface temperature) may differ from observations. Balancing this risk is the benefit that, having not used the observed climate record to aid in model tuning, we can genuinely use this record to evaluate our model. This is not the case for models that do use observed historical records and trends in their development and tuning.

We applied one important constraint in our model development: that the total anthropogenic effective radiative forcing (total ERF) for the PD (defined as year 2000) was required to be positive relative to the PI (1850) forcing. This is considered “certain” by IPCC AR5 (Myhre et al., 2013). An additional subsidiary constraint was also included; if the total ERF, or any of the component ERFs (e.g., due to greenhouse gases, aerosol, and land use), were significantly different from our predecessor models, HadGEM2‐ES (Collins et al., 2011) or HadGEM3‐GC2 (Williams et al., 2018), then we would investigate reasons for these differences. If nothing was found in error at the component level, then the large ERF differences would be accepted. A significant difference with respect to Year 2000 minus 1850 global mean ERF was defined as ±0.4 W m^−2^. Once the trunk model, HadGEM3‐GC3, had been developed, based on AMIP and fixed‐forcing PI and PD experiments, we performed a set of ERF simulations using its atmospheric component GA7 (Walters et al., 2019). These followed standard ERF experiment protocols (e.g., Andrews, 2014; Pincus et al., 2016), allowing us to diagnose the total anthropogenic ERF as well as the various component ERF terms. As documented in Table 1 of Mulcahy et al. (2018), the GA7 total ERF for Year 2000 minus 1850 was −0.6 W m^−2^. This was deemed unacceptable both for the physical model trunk HadGEM3‐GC3 and for UKESM1, with respect to their application in CMIP6. The primary cause of this negative total ERF was determined to be an excessively negative net aerosol ERF value of −2.75 W m^−2^ (Mulcahy et al., 2018). Of the historical ERF components that were constrained in IPCC AR5, the net aerosol ERF was the only GA7 value outside the 5–95% probability range, quoted as −1.9 to −0.1 W m^−2^ for the net aerosol ERF. To address this large discrepancy, processes controlling the model's aerosol ERF were analyzed and new aerosol and cloud microphysical processes introduced. Model developments were evaluated through running repeat AMIP and ERF experiments, allowing both the aerosol ERF and the overall model simulation quality to be monitored. This work is documented in Mulcahy et al. (2018) and resulted in a net aerosol ERF of −1.45 W m^−2^ and a total anthropogenic ERF of +0.75 W m^−2^, which satisfied both our primary and subsidiary constraints on ERF. The addition of these developments to GA7 led to a new atmospheric model branch, GA7.1, and its coupled counterpart, HadGEM3‐GC3.1, which formed the physical model core of UKESM1.

## 5 Characterization of Model Performance

The papers in this special issue are intended to describe, evaluate, and characterize the U.K. models for CMIP6. The results are so far largely limited to experiments that make up the CMIP6 DECK (Eyring et al., 2016; AMIP, piControl, 1%CO2, abrupt4xCO2, and the CMIP6 historical simulations), although use has also been made of historical radiative forcing experiments in RFMIP and CFMIP. A number of papers focus on developing and delivering the models and evaluation of PD performance against a wide range of diagnostics and metrics (Kuhlbrodt et al., 2018; Menary et al., 2019; Mulcahy et al., 2018; Sellar, Walton, et al., 2019; Williams et al., 2018, 2020) others include or focus on evaluation of historical trends (Andrews et al., 2020; Sellar, Jones, et al., 2019) or focus on understanding the forcing, feedbacks, climate sensitivity, and response (Andrews et al., 2019; Bodas‐Salcedo et al., 2019; Hardiman et al., 2019). Finally, Yool et al. (2020) documents the spin‐up procedure for UKESM1.

**Figure 1:**
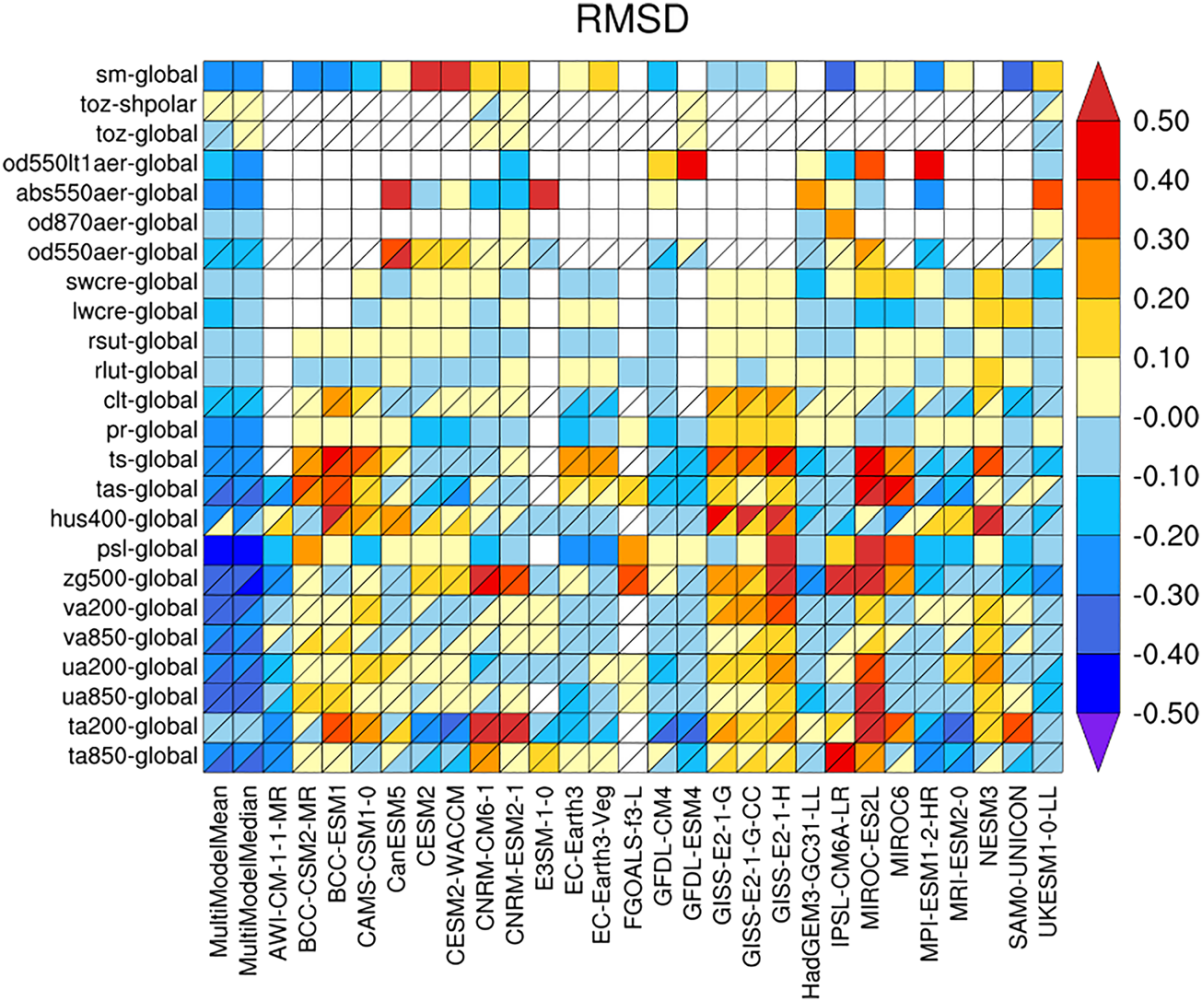
Portrait diagram displaying relative RMS error metrics for twentieth century CMIP6 annual cycle climatology (1997–2010)—following Gleckler et al. (2008). Shades of blue indicate cases where a model performs better than the typical model with respect to the reference data (as detailed by Gleckler et al., 2008), and shades of red the contrary. Where a grid square is split by a diagonal, the relative error is shown with respect to both the primary (upper left triangle) and the alternate (lower right triangle) reference data sets. In nonsplit grid squares only the primary reference data set is used.

The suite of U.K. models reproduce the PD climate with a high degree of fidelity, both with respect to standard fields and more process‐based measures of evaluation. In most fields the physical model HadGEM3‐GC3.1 performs better than HadGEM2‐AO (Martin et al., 2011) and HadGEM3‐GC2 (Williams et al., 2015), models that evaluated among the best of the CMIP5 multimodel ensemble. We use the “portrait” plot of relative root‐mean‐square (RMS) error (Gleckler et al., 2008) to illustrate that when measured over a range of atmospheric variables for the period 1997–2010, UKESM1 and HadGEM3‐GC3.1‐LL overall evaluate better than most other CMIP6 models for a broad range of variables (blue shading in Figure  1). The ability of both models to reproduce the historical temperature record (Andrews et al., 2020) lies within the range of models within CMIP5, although there are some clear discrepancies. In common with most CMIP5 models the warming from 1990 to PD is larger than seen in the observations, and both models show too strong a cooling during the period 1950–1980. Factors contributing to these discrepancies are briefly discussed in Andrews et al. (2020) with the role of aerosols being important (e.g., Archibald et al., 2019; Mulcahy et al., 2019; O'Connor et al., 2020).

**Figure 2:**
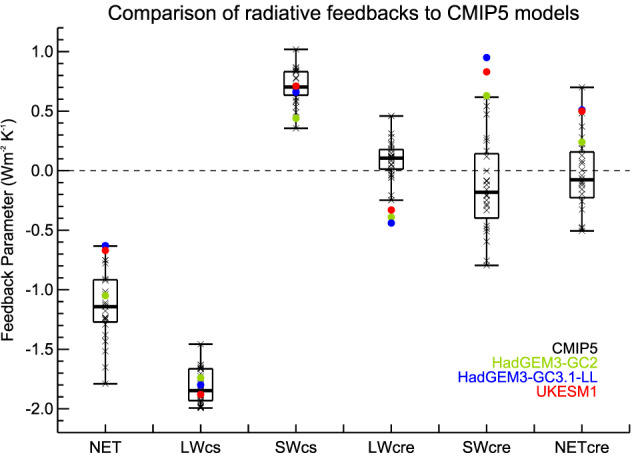
Global mean radiative feedback components in W m^−2^ K^−1^ derived from the 4xCO_2_ experiments for HadGEM3‐GC2, HadGEM3‐GC3.1, and UKESM1 and compared to the CMIP5 multimodel ensemble. The feedbacks are (from left to right) total (NET) feedback, longwave clear‐sky, shortwave clear‐sky, longwave cloud radiative effect (CRE), shortwave CRE, and net CRE. The box plots show the range (maximum and minimum), interquartile range, and median values of the feedbacks for the CMIP5 ensemble (crosses). Feedback parameters are calculated following Andrews et al. (2012), that is, from the slope of the linear regression of the change in radiative flux against deltaT for the 150 years of the 4xCO_2_ “step” experiment. The CMIP5 data are calculated in the same way, updated from Andrews et al. (2012) and Forster et al. (2013)

The EffCS of both HadGEM3‐GC3.1‐LL (and ‐MM) and UKESM1 are described in more detail in Andrews et al. (2019) and Sellar, Jones, et al. (2019) and are at the top end of the IPCC AR5 likely ranging and higher than any CMIP5 models (Table 2). Andrews et al. (2019) give a detailed breakdown of the feedbacks that sum up to produce the overall climate sensitivity. Figure 2 shows a summary of the net, clear‐sky, and cloud radiative feedbacks in UKESM1 and HadGEM3‐GC3.1‐LL compared to HadGEM3‐GC2 (Senior et al., 2016) and the CMIP5 ensemble. As discussed in Andrews et al. (2019), the net feedback in the new models is toward the top end of (but not outside of) the CMIP5 range. The EffCS lies outside of the CMIP5 range due to this feedback combining with a relatively “normal” sized forcing.

**Table 2:** Effective Climate Sensitivity (EffCS), Transient Climate Response (TCR), and Transient Climate Response to Cumulative Emissions (TCRE) in U.K. Models for CMIP6, as Documented in Andrews et al. (2019) and Sellar, Jones, et al. (2019).

Model	EffCS	TCR	TCRE
HadGEM3‐GC3.1‐LL	5.5	2.5	
HadGEM3‐GC3.1‐MM	5.4	2.7	
UKESM1	5.4	2.8	2.6

*Note. *EffCS is calculated following Andrews et al. (2012), that is, as half of the intercept on the deltaT axis of a linear regression of the change in radiative flux against deltaT for the 150 years of the 4xCO_2_ “step” experiment. TCR is the deltaT at the point of CO_2_ doubling (Year 70) in the 1% CO_2_ simulation, calculated as the mean over Years 61–80, and TCRE is TCR divided by the cumulative compatible anthropogenic emission in UKESM1 up to Year 70 of the 1%CO_2_ experiment.

Breaking down the net feedback highlights the role of both a change in the clear‐sky shortwave feedback (CSSW) relative to HadGEM3‐GC2 and an increase in the net cloud feedback, although in both cases these lie within the CMIP5 ensemble. The CSSW feedback increases largely due to improved (increased) sea ice coverage in HadGEM3‐GC3.1‐LL relative to HadGEM3‐GC2. Although the net cloud feedbacks lie within the CMIP5 ensemble, both HadGEM3‐GC3.1‐LL and HadGEM3‐GC2 show LW and SW component of cloud feedback that lie outside of the CMIP5 range. This is discussed in Senior et al. (2016) and is attributable to large (but compensating in LW and SW) changes in high cloud across the tropical Pacific. Work to evaluate the validity of such changes is ongoing. Bodas‐Salcedo et al. (2019) investigate the reasons for the changes in feedbacks between HadGEM3‐GC3.1‐LL and its immediate predecessor, HadGEM3‐GC2 (Senior et al., 2016). They find that the inclusion of new or improved physical mechanisms, notably associated with cloud‐aerosol interactions and cloud microphysics, reduces the size of negative cloud feedbacks but also typically improves the evaluation of the PD climate and, where testable, of interannual variations as well. Thus, one of the main drivers of the high sensitivity in HadGEM3‐GC3.1 and UKESM1 is a positive cloud feedback, notably over the subtropical oceans. This feedback was present, of similar magnitude, in earlier U.K. model versions (and in many other CMIP5 models) but was previously masked by balancing negative cloud feedbacks. These latter feedbacks may have been unrealistic, resulting from a poor simulation of PD, midlatitude clouds and have been significantly improved in HadGEM3‐GC3.1 and UKESM1. Finding observational constraints for the veracity of the remaining positive cloud feedbacks is hence a matter of urgency for the research community. The upcoming World Climate Research Council (WCRP) assessment of climate sensitivity may provide such a framework. Due to the current lack of such well‐founded constraints, we see no reason to tune parameters in our model simply to deliver a lower climate sensitivity and prefer to follow a model development pathway based on our best ability to simulate the key processes at the process level.

A perturbed parameter ensemble based on a model almost identical to HadGEM3‐GC3.1 delivers a limited range of climate sensitivity (4.2 to 6.2 K Rostron et al., 2020) as evidenced by the climate projections discussed in Murphy et al. (2018). Rostron et al. (2020) describe how filtering of a much larger ensemble of AMIP simulations based on evaluation criteria eliminates model versions with negative feedbacks suggesting that there is a structural constraint in the model whereby low values of EffCS are associated with a model climatology that is unacceptable. The standard or unperturbed version of the model sits at the upper end of the sensitivity space but also shows levels of agreement with observations that are hard to beat in any of the perturbed members that make up the 20‐member ensemble.

**Figure 3:**
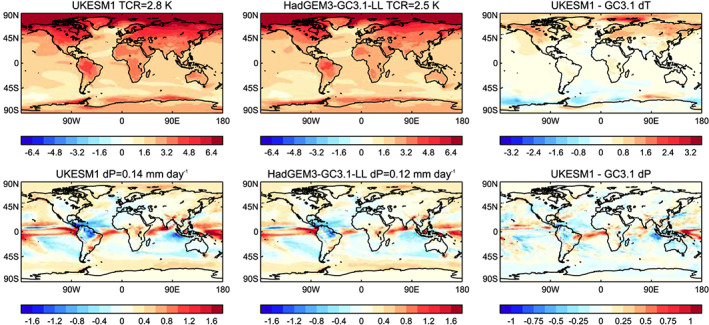
The top row shows the maps of TCR, and the bottom row shows the change in precipitation at the time of TCR (i.e., averaged over Years 61–80 of the 1%CO_2_ run). They are the average of four ensemble members of UKESM1 and HadGEM3‐GC3.1 1%CO_2_ runs.

Detailed analysis of the future projections will be the subject of papers beyond this special issue, but we note that the TCR and precipitation response averaged around the time of the TCR appear relatively insensitive to both changes in resolution and the inclusion of ES processes (Andrews et al., 2019), although there are some differences in the patterns of response to idealized forcing (Figure 3). These differences are consistent with the impact of biogeochemical processes as described in Andrews et al. (2019).

## 6 The Broader Modeling Landscape

Emerging results from CMIP6 suggest that a number of other models also exhibit climate sensitivities above the top of the CMIP5 ensemble (e.g., Forster et al., 2019; Gettleman et al., 2019; Golaz et al., 2019; Meehl et al., 2020; Swart et al., 2019), and there is now an urgent need to understand the reasons for this increase in sensitivity and whether there are any common reasons for this across the models. Meehl et al. (2020) provide a historical context for interpreting EffCS in the CMIP6 ensemble, describe the relationship to TCR, and review possible reasons for increased values from CMIP5. They find that cloud feedbacks and cloud‐aerosol interactions in particular, as described here for HadGEM3‐GC3.1 and UKESM1, are the most common contributors to this increase although there is no single cause in all cases. Zelinka et al. (2020) break down the cloud radiative feedback across the CMIP6 ensemble, finding stronger positive shortwave cloud feedbacks from decreasing extratropical low cloud coverage and albedo, with CMIP6 models showing weaker increases in extratropical low cloud cover and water content with warming SST. Again, these results closely align to our own findings with HadGEM3‐GC3.1 and UKESM1 (Bodas‐Salcedo et al., 2019). There appears to be emerging evidence that the high EffCS in many models is due to the removal of a negative shortwave cloud feedback in earlier models associated with an increase in cloud water content, often linked to a warming atmosphere seeing large‐scale conversion of cloud ice to cloud liquid water. This feedback is smaller in some CMIP6 models, due to the introduction of a more realistic representation of supercooled cloud liquid water (Tan et al., 2016) limiting this phase change feedback and thereby exposing a positive shortwave cloud feedback that results in an increased positive net cloud feedback.

It is clear that the higher EffCS values from some new‐generation models will have important implications for carbon budgets and adaptation time scales and will mean an increased risk of passing climate tipping points—such as the thawing of permafrost—which may further accelerate warming (Forster et al., 2019). There is therefore an urgent need to assess the plausibility of the more positive net cloud feedbacks, in particular, the positive SW feedbacks that have been present in both CMIP5 and CMIP6 models. The upcoming WCRP assessment of climate sensitivity, using multiple lines of evidence to deliver a new constraint on EffCS and a framework for testing models at the process level, will be a valuable tool in this respect. We believe it is critical that all models are tested in this way, not simply models with global mean temperature trends that fall outside of the current and future assessed “likely” range. Our experience is that models which fall within these ranges may well do so through compensating biases and hence do not necessarily provide more reliable projections of patterns of temperature response, critical aspects of the hydrological cycle, or emerging signals of forced climate change over natural climate variability.

## 7 Summary

This paper highlights the new approach the United Kingdom has taken to delivering a second‐generation ES model for CMIP6, through a joint activity between the Met Office and the U.K. NERC/academic community. We describe how a jointly funded core team dispersed across many centers, but with a critical mass based at the Met Office, has been able to pull through new representation of ES processes developed at many institutions into UKESM1, while building on parallel developments of the core physical model, HadGEM3‐GC3.1, made through the Met Office seamless Unified Model system. We document our approach to model tuning for CMIP6 and the extensive methods used in model evaluation, highlighting papers within this special issue that extend the analysis reported here. We briefly characterize the performance of HadGEM3‐GC3.1 and UKESM1 based on their respective CMIP6 DECK simulations (Eyring et al., 2016) referencing many more comprehensive studies within this special issue. The emergent properties of the models, notably the high climate sensitivity, are of particular interest. We discuss our initial findings, again pointing to papers in this special issue that describe the systematic approach we have taken to understanding the key drivers of this increase in EffCS since previous model versions were released. We also provide some context of the broader modeling landscape in CMIP6 and how understanding of the drivers of increased sensitivity in HadGEM3‐GC3.1 and UKESM1 appear to align with a number of other CMIP6 models. Finally, we argue that the international modeling community must rapidly find new observation‐based process‐level constraints for aspects of cloud feedback which appear, for at least some models, to be a common cause of the increase in EffCS since CMIP5. Critically, we feel all models, regardless of whether their EffCS falls within or outside of assessed “likely” ranges, should be tested at this process level. Experience has taught us that compensation of biases in models that sit within this “likely” range does not mean they should be given more weight as delivering plausible projections without passing such process‐level evaluation.

## Acronyms

NCAS: National Centre for Atmospheric Research
BAS: British Antarctic Survey
BGS: British Geological Survey
CEH: Centre for Ecology and Hydrology
CPOM: Centre for Polar Observation and Modelling
NCEO: National Centre for Earth Observation
NOC: National Oceanography Centre
PML: Proudman Marine Laboratory
JWCRP: Joint Weather and Climate Research Programme
NERC: National Environmental Research Council
CEDA: Centre for Environmental Data Analysis
WCRP: World Climate Research Council
NWP: Numerical Weather Prediction
MIP: Model Intercomparison Project
PEG: Process Evaluation Group
AMIP: Atmospheric Model Intercomparison Project
AOIL: Atmosphere‐Ocean‐Ice‐Land
GLOMAP: GLObal Model of Aerosol Processes
DECK: Diagnoses, Evaluation and Characterization of Klima

## Data Availability

HadGEM3‐GC3.1 and UKESM1 data are being made available from the CMIP6 data archive (https://cmip-pcmdi.llnl.gov/cmip6/).
